# Out of the Mouths of Babes: Black Children’s Experiences of Emotion-Focused Racial–Ethnic Socialization, Coping, and Antiracist Resistance

**DOI:** 10.3390/bs15020222

**Published:** 2025-02-16

**Authors:** Emilie Phillips Smith, Simone E. Bibbs, Deborah J. Johnson, Lekie Dwanyen, Kendal Holtrop, LaVelle Gipson-Tansil

**Affiliations:** Department of Human Development and Family Studies, Michigan State University, East Lansing, MI 48824, USA; bibbssim@msu.edu (S.E.B.); john1442@msu.edu (D.J.J.); dwanyenl@msu.edu (L.D.); holtropk@msu.edu (K.H.); gipson@msu.edu (L.G.-T.)

**Keywords:** racial–ethnic socialization, racial–ethnic identity, cultural socialization, preparation for barriers, discrimination, children, parents, caregivers, black children and families

## Abstract

Black children in the U.S. learn from scaffolded parental teachings to help manage racial discrimination. Middle childhood is an understudied developmental period for this research. This paper builds upon research on culturally informed practices Black caregivers use to rear their young with a healthy identity and socio-emotional skills to navigate racism Guided by a phenomenological qualitative approach, we conducted focus groups with 39 Black children (Mean_age_ = 7.67, 54% girls, 46% boys). Children reported that their parents imparted a sense of positive identity in terms of their cultural heritage, skin, and hair—areas in which they experienced frequent bullying. A uniqueness of our study is that Black children also reported learning emotion-centered coping strategies that focus on their inner strengths and private speech. They adopted a range of adaptive coping mechanisms such as kindness, ignoring perpetrators, centering their positive identity, *identity framing*, and fighting back. Through children’s voices, we build upon previous research integrating racial–ethnic socialization (RES) with socio-emotional competencies in response to discrimination. We underscore the importance of exploring racial–ethnic identity development and socialization in childhood, a developmental period in which these processes are understudied.

## 1. Introduction

Black children in the United States (U.S.) learn to decipher and navigate racialized interactions through parental teachings intended to help them manage discrimination and threats to their well-being. This paper builds upon research on culturally informed practices Black caregivers use to rear their young with a healthy identity and socio-emotional skills to navigate racism (L. A. Anderson et al., 2022; R. E. Anderson et al., 2024; Banerjee et al., 2017; E. P. Smith et al., 2022). Previous studies examining racial–ethnic discrimination indicate that Black youth frequently experience discrimination in multiple aspects of their lives (Cave et al., 2020; Seaton & Iida, 2019). Black and Brown children as young as age seven describe a range of prejudice experiences that range from stereotyping their character and abilities, to being overly policed when shopping in stores or in their communities (Dulin-Keita et al., 2011). Discrimination has an enduring impact on developing youth and is associated with higher levels of stress, substance use, and behavioral and mental health problems such as depression and anxiety (Brody et al., 2006; Cave et al., 2020; McNeil Smith & Gobin, 2024; Neblett et al., 2008; Sellers et al., 2006). Black children and their families contend with these regular threats to their identity and well-being in their daily life.

So much of the research on identity and discrimination has focused on adolescent and adult populations. Yet, the research reveals that racial–ethnic identity begins to crystallize as early as ages 9 or 10 (elementary school) and is related to a range of child- and parent-reported academic and behavioral outcomes, including substance use risk (Augustine et al., 2022; Chavous et al., 2008; Rivas-Drake et al., 2014; C. Smith et al., 2009; Yu et al., 2021). A longitudinal study of 642 African American children from kindergarten to third grade revealed relationships of racial–ethnic identity in middle childhood to their self-esteem, parental reports of their social competence, and standardized tests of cognitive and academic learning (C. Smith et al., 2009).

While a positive sense of racial–ethnic identity has also been found to be important in reducing depression and stress among adolescents, the research is mixed on the degree to which even a positive identity is sufficient to buffer the effects of discrimination (Brody et al., 2006; Sellers et al., 2006; Seaton & Iida, 2019). Cave et al. (2020) found that the effects of discrimination upon health and mental health outcomes were particularly deleterious for children aged 5–10 in comparison to the effects upon children aged 11–18. Though studies in middle childhood were only 20 percent of the 88 identified studies examining the effects of racism, studies with younger elementary-aged children were more likely to demonstrate adverse effects of exposure to racism on problem behaviors such as aggression, alcohol or drug use. In a study of a subsample of Black children from the National Survey of Children’s Health (M_age_ = 9.8 years), discrimination was found to be a powerful adverse childhood experience (ACE) and significantly related to mental health, even after controlling for other traumatic experiences such as domestic violence, abuse, and parental divorce (Bernard et al., 2022). These findings suggest that racism is traumatic, and it is unreasonable to expect individual youth resilience to suffice alone; the consistent adverse effects of discrimination point to the need for more structural approaches to addressing racism (Bonilla-Silva, 2023).

In the U.S., many caregivers of Black children endeavor to prepare them for the racism and discrimination they will inevitably encounter. Decades of research has examined dimensions of racial–ethnic socialization (RES) intended to foster positive identity and development in the face of interpersonal and systemic racism (Caughy et al., 2002; Hughes et al., 2006; Neblett et al., 2008; Peters, 1985; E. P. Smith et al., 2003; Umaña-Taylor & Hill, 2020). Messages focused on the positive meaning of one’s cultural heritage and identity have been found to be the most helpful in fostering racial–ethnic identity and competence.

Preparation for racial barriers is another RES strategy; research on this approach has grown substantially, evidencing mixed effects. In some research, these approaches are related to elevated levels of anger, stress, and depression (Banerjee et al., 2015; Elmore & Gaylord-Harden, 2013; Stevenson, 1998; Stevenson & Arrington, 2009). Anger or sadness in the face of discrimination is understandable given how frequent, pervasive, and early these experiences emerge. Some research with Black adolescent boys demonstrates that even RES has limitations in ameliorating the impact of discrimination on children (Khahra et al., 2024; Saleem & Lambert, 2016; Varner et al., 2018).

Yet, other research has found that awareness of racial discrimination and bias can co-exist with a healthy sense of racial-identity and other aspects of positive youth development (Umaña-Taylor & Hill, 2020; Yu et al., 2021). In a latent profile analysis of African American and Latine elementary-age children, both the “proud and optimistic” group (high in racial pride, lower in perceived barriers) and the “proud and aware” group (high in racial pride and perceived barriers) demonstrated higher standardized scores in math and reading achievement than “disconnected” youth who did not express a positive sense of identity (Yu et al., 2021). Ultimately, scholars surmise that Black children must balance optimism in navigating the world while acknowledging some awareness, anger, and anxiety as reactions to discrimination (Stevenson, 1998). Indeed, this balance of emotions could fuel radical hope and antiracist resistance on the part of developing children.

Preparing children for bias involves more complex processes. For example, in families with quality relationships (i.e., trust and communication), adverse effects of preparing children for racial bias are not evident (Lambert et al., 2015). Emotion socialization has potential as an integral part of those protective messages for Black American children, in which parents validate and help children to understand and manage their emotional responses to bias and discrimination (Dunbar et al., 2017). Dunbar et al. (2017) postulate that parental scaffolding in both the content and process of communicating racial socialization messages might help ameliorate some of the detrimental effects of discrimination upon children’s mental health. For example, parental emotion-focused racial socialization, which integrates RES with practices that teach emotional understanding and regulation, has been found to help young people cope with the effects of discrimination. Importantly, this research has found some variation in parenting RES based upon gender. Mothers who expressed more warmth with girls who had a pattern of “submissive reactivity” (i.e., embarrassment or sadness) were found to disrupt the linkages between discrimination and internalizing/externalizing behavior. For boys, a more “no-nonsense” approach reduced this submissive reactivity of shame and sadness (Dunbar et al., 2022). Helping adolescents manage their responses to acts of exclusion is important to their mental health.

Socio-emotional learning involves elements of self-awareness, self-management, and responsible decision making, in addition to managing relationships and social awareness (Greenberg et al., 2017; Greenberg, 2023). Exploring the socio-emotional development of children resonates with the classic conceptual work of Vygotsky (1978, 1934/1987). Vygotsky viewed parenting as part of a transactional cultural exchange between parents and children, attuned to the child’s role in the exchange. Vygotsky explored children’s *private speech*, that is the ways in which they talked to themselves about who they are and their human potentiality. Vygotsky’s ideas might connect to more current conceptualizations of private and public regard in racial–ethnic identity research. *Private regard* refers to perceiving value in one’s own racial–ethnic heritage, regardless of *public regard*, how larger society might attribute stereotypical and unfavorable views to one’s racial–ethnic background (Sellers et al., 1997). Development of a positive private regard is a central task for minoritized youth (Davis et al., 2017). In terms of socialization, Vygotsky regarded the parent as a “more knowledgeable other” (MKO), who helps the child navigate and reach more success than is possible without their invaluable scaffolding. Other examples of the MKO examines the role of mentors for Black boys, who provide support in socio-emotional regulation, buffering the experiences of school-based discrimination (Khahra et al., 2024). In the wake of discriminatory experiences, the MKO is a pivotal agent in fostering socio-emotional regulation that can help developing children identify their experiences, the range of potential reactions, and make choices that are helpful and healthy to their own adjustment.

In summary, over the past few decades, research has examined the relationship between RES and discrimination, mental health, and health outcomes, most often with adolescent or college populations (R. E. Anderson et al., 2024; Benner et al., 2018; Umaña-Taylor & Hill, 2020; Wang et al., 2020). Exploring younger children’s perceptions of RES acknowledges racial–ethnic identity as a developmental process that is salient at early ages and related to important socio-emotional and academic outcomes (C. Smith et al., 2009). Furthermore, exploring children’s perceptions of parental messages begins to honor their role in the interactive, transactional process of RES. Children engage with their parents around key issues of the meaning of their race, how others might interact with them, as well as how and when they should personally respond in ways that foster a positive sense of self, adaptation, and success in their proximal environments.

Understanding RES among Black parents informs next-generation prevention science approaches attuned to effective ways to support minoritized families in the work of parenting, emotional, and racial–ethnic socialization (Brody et al., 2021; Caldwell et al., 2014; Murry et al., 2023; E. P. Smith et al., 2022; Stein et al., 2021). Though few and far between, parenting programs specifically developed for Black families, that integrate attention and the processes of promoting racial socialization in middle childhood (age 11), have been found to enhance children’s later academic competence in middle adolescence (age 15) (Murry et al., 2023). Among immigrant Latine families, attention to the ways in which parents socialize their children in the contexts of discrimination and documentation challenges has been found to yield superior results on mental health than a focus on parenting alone (Parra-Cardona et al., 2017). Increasingly, social media and online spaces serve as prominent contexts for intense and frequent racial discrimination among Black youth (English et al., 2020; Tynes et al., 2015). The complex tasks of preparing and protecting Black children from discrimination involves fostering a positive sense of culture and heritage, yet equipping them emotionally to grapple with and appropriately respond to racism and microaggressions.

The objective of the current study, the Family Voices Project, was to inform prevention science on how best to integrate parenting, social skills, and adaptive racial coping, particularly in a changing racial context and rising social media exposure. Thus, we sought input from parents and their elementary-age children to inform the development of equity-based, culturally relevant approaches to family-based prevention (E. P. Smith et al., 2022, 2023). The study described in this paper was conducted as part of a broader project exploring parenting strategies that foster their children’s positive social, educational, and future occupational experiences. Specifically, the investigators sought to understand Black parents’ experiences with education, work, and how they inform racial–ethnic socialization strategies shared with their children. Most importantly, this inductive study was designed to honor the voices of children by exploring ways in which parents and children broach the topics of race, identity, and coping with discrimination.

## 2. Materials and Methods

### 2.1. Research Design

Given the present study’s goal of advancing culturally relevant family programs for Black families, our research questions, methods of data collection and analysis, and decisions related to inferences and use of study findings were guided by a transformative paradigm—a social-justice-oriented research framework that argues for the construction of knowledge to transform the lives of marginalized individuals and communities by centering their experiences, reducing disparities, and “link[ing] the results of social inquiry to action” (Mertens, 1999, p. 4). The current paper draws from a larger phenomenological study exploring parenting strategies that foster Black children’s positive social, educational, and future occupational experiences. Phenomenology aims to “make meaning” by gathering the collective experiences of a phenomenon. Using a descriptive phenomenological approach (Husserl, 1964), we aimed to understand Black children’s meaning making around RES experiences, including racial discrimination and coping mechanisms they employ to resist racism and discrimination.

While focus groups with adults and adolescents are more frequent, focus group research with children is more novel and recognizes their developing levels of identity, agency, and self-knowledge in the transactional parent-child-parent socialization process (Hughes & Chen, 1997; Smith-Bynum, 2023; Vygotsky, 1978, 1934/1987). In the current research, using child focus groups offers a rare perspective drawing upon innovative methods involving not only conversations with groups of children, but also artistic expressions that were expected to facilitate conversation, and help them discuss difficult topics, in ways that demonstrate the developmental competencies of children (Duane, 2023; Jackson Foster et al., 2018; Jones & Broome, 2001; Stacy et al., 2018).

### 2.2. Research Participants

Participants in this study included 39 Black children aged 5–12 years old (Mean_age_ = 7.67 years; 54% girls, 46% boys). Families were recruited via flyers shared through schools and community-based afterschool programs. Caregivers interested in participating in the current study were instructed to scan a QR code to access the online eligibility screening survey. Adults were considered eligible if they identified as Black and were the primary caregiver of a Black child (or children) aged 5 to 12 years old. Eligible caregivers were prompted to read a consent form, enabling them to make an informed decision about whether they wanted to participate in the research project and whether they wanted to allow the participation of their child(ren) in this study. Ineligible survey respondents were redirected to a webpage providing information about local summer programs for kids.

Eligible families were contacted by research team members via phone or email to schedule a focus group date. Upon arriving at the focus group site, consent forms were provided to caregivers with their child present, and a trained research assistant reviewed the study information. Informed consent was obtained from caregivers and informed assent from their children. Children were only permitted to participate in a focus group if both were acquired. Prior to each session, families were provided with a meal and the opportunity to share a communal space with other participating Black families. Following dinner, caregivers and children participated in separate focus groups. Five child focus group sessions were conducted in total. Facilitators began each child focus group by explaining the goal of the discussion. Children were reminded that their participation was voluntary, and if they did not wish to participate in the group discussion, a research assistant could provide them with art materials and escort them to their caregiver(s). Caregivers received financial compensation for participation in the parent focus groups, and children were given stickers and small toys for their study participation. All study procedures received approval from the university Human Research Protection Program.

### 2.3. Research Context

Data collection took place in a small Midwestern city in the United States at three locations: a local YMCA, a university-run child development lab, and an independent Black school providing after-school programming. It was imperative that our focus groups were conducted in community-based locations that afforded safe, comfortable, and confidential conversations among parents, caregivers, and their children. Family meals, child-care for younger children, and transportation were provided to foster participation. Research sites were intentionally chosen to build trust, reduce barriers to participation, foster more authentic interactions, and empower participants by valuing their knowledge and experiences.

### 2.4. Positionality and Reflexivity

Our data collection team was comprised of Black faculty, undergraduate and graduate students, and community members, as well as one White faculty member with parenting program expertise. Two faculty members were systemic family therapists with clinical expertise working with parent–child dyads and access to referrals for families requiring additional services.

The coding team for the current analysis consisted of two senior scholars, one graduate student researcher, and one undergraduate research assistant. Our positionalities as African American women, students, and scholars locate us in relation to our participants as educators, listeners, and corroborators. Two members of our team are mothers who have parented Black children, and we all grew up as Black children navigating unique school and neighborhood contexts. Along with our White colleagues collaborating on this work, we as researchers are committed to scholarship that positively impacts the lives of marginalized individuals and families, particularly attuned to the ways racism is embedded within U.S. social structures and institutions and to the ways Black youth must navigate contexts that are racialized, discriminatory, and derogatory. In short, we recognize the role of culture and heritage in the interpretation of data, with a keen awareness of the power dynamics in our research and society that marginalize Black children (Rodriguez et al., 2011). Yet, our work is driven by the principle conveyed by Article 12 of the United Nations Convention on the Rights of the Child (United Nations, 1989), that “children have the right to give their opinions freely on issues that affect them,” in concert with the adults who are responsible for their care.

True to all data analysis, we acknowledge that the interpretations of the stories and experiences shared in the current study may be influenced by our own personal experiences, beliefs, and philosophies. However, in line with phenomenology practice, intentional efforts were made to mitigate personal biases and ensure that our interpretations were supported by the data collected (Finlay, 2014). One such effort included sharing preliminary findings with members of a larger research group throughout the research process. Inviting insight from diverse perspectives helped ensure our findings reduced bias in their reflection of participants’ experiences (Willis et al., 2016). Along with sharing findings with individuals external to our coding team, we also engaged in reflexivity practices as data were analyzed. While in group meetings, we actively reflected on our subjectivities and biases and discussed how our personal backgrounds and past experiences may influence our interpretation of the data—key processes for enhancing rigor in qualitative research (J. L. Johnson et al., 2020).

### 2.5. Data Collection and Analysis

Each focus group began with an introduction to the study by the facilitators and a description of the activities to take place during the session. Before formally initiating each focus group, the facilitator and participants co-created guidelines for the discussion. The facilitators began by sharing a rule (e.g., do not talk over someone while they are speaking) and then asked participants to voice additional rules they thought might be important to follow while talking together as a group. Once group rules were established, focus groups commenced. Along with two facilitators, a research assistant was present to take note of environmental cues and document participants’ non-verbal responses to the interview questions. A semi-structured interview guide was used to pose open-ended questions assessing children’s experiences of RES and discrimination. These questions included the following:

1. What has your family taught you about what is awesome or great about being a Black girl or Black boy?
2. What has your family told you is hard about being a Black girl or Black boy?
3. Have you ever been picked on, made fun of, or treated unfairly because you’re Black (for example, teased because of your skin color or hairstyle)?
4. What has your family taught you about what to do when you feel picked on, made fun of, or treated unfairly because you are Black?

Focus groups averaged approximately 52 min in duration. All focus groups were audio recorded and then transcribed using a professional transcription service. To gain a comprehensive understanding of children’s experiences, data analysis was guided by thematic analysis protocols (Braun & Clarke, 2006). After deidentifying our transcripts, we began the first phase of the analysis—familiarizing ourselves with the data. This phase involved reading and rereading our transcripts and meeting as a group to discuss our initial insights. During phase two of the analysis, team members individually completed open coding of the transcripts, highlighting significant statements, and labeling them with a descriptive code (referred to as “horizontalization” (Creswell & Poth, 2018)). During phase three, the team reconvened as a group to discuss and chart our initial codes, as well as identify potential themes or “clusters of meaning” (Moustakas, 1994). Phase four of the analysis involved going back to the transcripts individually to gather all data into the themes generated during phase three. Following this, phase five entailed meeting again as a group to review our themes and verify that they worked in relation to our data; themes were refined, combined, or removed accordingly. Names and descriptions for each theme were also created. After clearly defining and naming our themes, we proceeded to the final phase of analysis—formally writing our findings and selecting quotes from our data that most vividly captured the essence of the lived experiences shared among our participants.

## 3. Results

From our findings, we identified four themes related to children’s experiences of RES and discrimination and their responses to race-based bullying: (1) *Black cultural pride and heritage*; (2) *preparation for bias and discrimination*; (3) *microaggressions and anti-Black racism from peers*; and (4) *socio-emotional responses to race-based bullying*, including a range of racial coping strategies. Some of the themes are areas identified in more than a decade of prior literature while others on emotion-focused coping represent more recently emerging areas. In the sections to follow, we describe our findings and provide exemplar statements in making meaning of children’s experience of what it is like to be a Black child in the United States, and their view of the ways in which their parents and families instill positive identities and racial coping.

### 3.1. Black Cultural Pride and Heritage

Building upon a strengths-based perspective of Black families (García Coll et al., 1996; Hill et al., 2023), we began our focus groups by asking children, “What has your family taught you about what is awesome or great about being a Black child?” Participants shared that their parents help them recognize their own unique characteristics to help them develop pride in their culture and heritage. For example, this process was evident in these responses from a six-year-old boy, ten-year-old-girl, and seven-year-old girl, respectively:

“My mom says since I have different blood and culture in me, I’m special, I’m amazing, I’m beautiful. I’m designed by God, so I must be special”.

“I’m Black and I’m proud”.

“My mom tells me every day that I’m smart and beautiful and I’ll be successful in life soon”.

Children in our focus groups also readily shared messages reflecting Black history learned from parents. A boy, aged six, and a girl, aged ten, stated the following:

“My parents told me that there was a Black kid named Ruby Bridges. That was the first African American to go to a White school”.

“So, they give me information about Black history like Martin Luther King and other people in the past. That helps me learn more about it”.

Another young girl, aged 12, explained the following:

“My grandma, [grandma’s name], she tells me that Black is beautiful, and that’s why she tells us all the time that we should look into our history because…, and also, it’s a blessing that we get treated differently now than back then because it’s crazy back then. But then she said Black is beautiful so that you should be proud of yourself and your color”.

Children reported how their caregivers, both parents and grandparents, were intentionally inculcating a positive sense of identity and cultural heritage. Families shared examples of young Black children like Ruby Bridges, who learned to navigate daily racism and persevere when she became the first African American student to integrate a formerly all-White elementary school in Louisiana (Michals, 2015).

### 3.2. Preparation for Bias and Discrimination

When asked what their families taught them about the difficulties of being a Black child, some of the lessons initially shared by participants were more generally about building character, messages of work, and responsibility, as well as difficulties of accomplishing these tasks as a Black person. Parents were often candid in sharing their own difficulties in life in the child-rearing process, as expressed in the following quotes from a seven-year-old girl and five-year-old boy:

“You have to pay bills and rent”.

“It’s hard to be a Black woman or a Black man that is taking care of kids”.

More messages on interpersonal preparation for bias were also reported by participants. For example, children were taught that they may experience mistreatment from peers in the forms of mocking and social exclusion, reflected in these responses from an 11-year-old girl and 6-year-old boy, respectively:

“A person that may not be of color could be talking about you behind your back to one of their friends because you’re darker than them”.

“…when somebody is playing a game, they might say you can’t play because of your skin color”.

Another girl, aged 11, stated:

“You might be teased or just ignored, and you might be asked a lot of questions”.

Along with preparation for bias from peers, children also received candid messages preparing them for bias more broadly. Contrary to raising their awareness of discrimination experiences that may happen, these messages from caregivers prepared children for experiences of discrimination that they were *certain* to occur within a white supremacist and normative system that marginalizes Black children. When asked what she was taught may be hard about being a Black girl, one 11-year-old girl simply stated:

“Other people won’t accept us”.

### 3.3. Microaggressions and Anti-Black Racism from Peers

When asked to recount their own experiences with racial discrimination and bullying, children reported microaggressions related to aspects of their physical appearance, as well as explicitly racist epithets from peers. Participants commonly described microaggressions related to their hair, especially among Black girls in our study. Despite its role as a central aspect of identity, self-expression, and self-esteem for many Black youth, hair can also be a site of trauma and constant negotiation (Mbilishaka & Apugo, 2020). Consistent with previous studies examining hair-based discrimination experiences among Black girls (Gadson & Lewis, 2022; Henning et al., 2022), Black girls in our focus groups reported verbal teasing, questioning, and unwanted physical touching of their hair. Two girls, both 11 years old, shared the following:

“There was this one time I got my hair box braided and there was this girl and she was making fun of me because of it. I don’t really know why, but she was making fun of me because of it”.

“People ask me why I would always wear an Afro, or ‘Can I touch it?’ and that kind of made me feel really uncomfortable…”

Including microaggressions, children also reported experiences of overt racism from non-Black school peers that most often took the form of derogatory name-calling. One 12-year-old girl described a shared experience among Black students attending her school:

“Some of all the kids basically would come up to the Black kids… they would call them monkeys and call us monkeys and call us slaves, and then they’ll be like, ‘Tie my shoes, you slave.’ or ‘Get my backpack”.

Another 10-year-old girl recalled an experience of being teased because of her skin color:

“So, there was these kids that were making fun of me and my friends because of our skin color. It was like they were just making fun of her skin and then telling us we’re ugly and stuff and just calling us names…like blackie…”

### 3.4. Socio-Emotional Responses to Race-Based Bullying

Black children are not a homogenous group and emanate from various family backgrounds that instruct them to respond in multiple ways to acts of race-based bullying. When it comes to responding to race-based bullying, we identified a range of emotion-focused coping strategies children learned from their caregivers including (1) *ignore the perpetrator*; (2) *show kindness;* (3) *retaliate*; (4) *report bullying;* (5) *emotion-focused coping* that helps them recognize and regulate their feelings; (6) *“identity framing”*, a culturally responsive strategy that centers on identity as an act of resistance; and (7) *constructive engagement* designed to inform and stop bullying behavior.

#### 3.4.1. Ignore the Perpetrator

Several children reported being told to ignore microaggressions and verbal denigration, particularly when parents perceived the behavior as less egregious, as evidenced by these responses from an eight-year-old girl and 11-year-old girl:

“My mom told me to just let it go. And she said what comes around goes around”.

“They’ll sometimes say just keep it rolling, don’t let other people talk about you, or you could just zone it out or ignore them”.

However, ignoring is a strategy to be used with care in that some children felt it was ineffective in addressing the situation. One 11-year-old girl’s experience with her stepmom illustrates this:

“So, my stepmom told me to ignore them, but it just got worser to worser things”.

#### 3.4.2. Show Kindness

Some children reported hearing messages from their caregivers encouraging them not to reciprocate bullying behavior; instead, caregivers emphasized character-building lessons of kindness and compassion, even towards a bully. Taught by her parents to reciprocate bullying behaviors with kindness, this seven-year-old stated the following:

“If someone be mean to you and you shouldn’t be mean back to them, you should always be kind. Even if they treat you as mean as they can”.

Another girl, six years old, shared a similar sentiment:

“At school, people might bully you because of your color, that doesn’t mean you bully them”.

#### 3.4.3. Retaliate

Despite some children learning to treat bullies with kindness in the hopes of a positive resolution, some children expressed that their parents felt very strongly that they should retaliate against bullying behavior swiftly and in-kind, particularly if this behavior was persistent, as was the case for this 12-year-old girl:

“They say just let it go. They don’t say let it go, but they do say don’t let them get to you, basically tell me to keep on going. Don’t let it get to you.. because if any situation gets serious, then..then, take matters into your own hands”.

#### 3.4.4. Report Bullying

Alerting parent(s) or teacher(s) about troublesome behavior was another strategy participants learned and employed, with a six-year-old girl affirming:

“If someone bullies you, go tell your teacher that you’re bullied”.

Although some participants were told to report bullying behavior, they did not always feel heard by their parents until the situation had escalated to an unacceptable point. In one case, a child described her daily experiences with bullying while on the bus to school. It was only after telling her parents multiple times about the bullying behavior that they moved her to a new school district:

“… I remember I told my mom first and she didn’t really know what to say because she never went through it. And then I told my dad, I guess they didn’t believe it was too bad, so they were like, ‘If it keeps happening, tell us.’ And I told him after eight times they finally took me out the school district…”

One young girl explained that she tries to handle the situation herself before informing her mother who she knows will be a swift and vocal advocate on her behalf, stating: “she’d go all crazy”. Interestingly, this 12-year-old girl referred to this approach as a way of protecting her mother from having to respond, while also saving the perpetrator from her mother’s ire.

#### 3.4.5. Emotion-Focused Racial Coping

During the focus groups, children were asked about how race-based experiences made them feel, describing a range of emotions including anger, annoyance, disappointment, sadness, discomfort, and indifference. To manage this wide range of emotional responses to negative racialized experiences, children reported strategies for racial coping that integrated helpful socio-emotional strategies. A seven-year-old girl and seven-year-old boy expressed the following, respectively:

“If someone just say something very rude to you that hurt your feelings, you just calm down and try not to think about it and do something calm or fun to get your mind out of it”.

“So, if a teacher bothers you, you might get mad and probably try to smack them in the face probably. But if you do that you’ll get in trouble. So, you have to just calm down and then be calm and then listen to the teacher”.

Warmth and emotional support from their families also helped support children’s self-regulation. Children reported receiving physical comfort from their caregivers to help them cope with and respond to the negative emotional effects of race-based bullying experiences, reflected in this statement from a six-year-old girl:

“Another thing that makes me calm is every time I hug my family, when I get mad, they make me calm. So, I go to sleep and then I get really calm so when I wake up I don’t have to be mad…”

Children’s reports also reveal that parents and caregivers sought to psychologically strengthen their youth by drawing upon cultural, spiritual, and religious beliefs to cope with adverse experiences (Stevenson, 1998). Specifically, they utilized cultural socialization strategies, designed to capacitate a sense of inner knowing, and antiracism resistance in their children. For example, during instances of race-based bullying, one 12-year-old girl found herself relying on an important lesson learned from her grandmother:

“But also, sometimes, I just remember what my grandma tells me. She tells me, ‘If you know you’re right, then just walk away’. And also, one of my favorite scriptures is ‘Let God take the wheel. Sit back and let God fight your battles.’ So I just walk off….They don’t say let it go, but they do say don’t let them get to you, basically tell me to keep on going”.

The strategies reportedly used by this grandmother draws upon aspects of cultural wealth (e.g., familial, navigational, resistant, among others) to help children cope in contexts of systemic racism (Yosso, 2005).

#### 3.4.6. Identity Framing

In conjunction with strategies for regulating emotions, children were taught to draw upon their own positive sense of identity, identity framing, in order to resist mistreatment from others. By teaching them to center their own thoughts and focus on what they know and believe about themselves, versus how others might attempt to portray them, parents used their children’s identities to lead their proactive approaches to addressing bullying. One nine-year-old boy described a process of cultivating an affirmed sense of self by ignoring negative comments from bullies and prioritizing his own more positive thoughts:

“They said don’t listen to the people that tell you stuff. Just listen to the nice stuff that you think of, and then think of your own ways to tell them how they are treating you”.

When asked how she responded to receiving unwanted questions about her hair, another child’s response reflected a similar process of reaffirming her unique identity as an act of resistance, sharing the following:

“…I would just ignore it and then think of myself as just special and myself alone”.

An 11-year-old female also exemplified the importance of focusing upon personal views of oneself versus the perceptions of others:

“I would also tell them that some people would treat them not as well because we have color. So yeah, and you should always be happy and don’t think of what other people think of you”.

#### 3.4.7. Constructive Engagement

We found that in some instances, in order protect their children and teach them to make good decisions about when and how to respond to bullies, children were encouraged to procedurally assess bullying situations. One 11-year-old girl shared her multi-step process for managing bullies:

“If somebody bullies you, ignore them. And then start step two if they don’t stop. Then, if they don’t stop then…step two was if they don’t stop, walk away. Step three was if they keep following you and doing that, then try until ignore them or go somewhere else. Step four, was go tell the teacher if it keeps happening”.

A six-year-old girl described greater ability to resist bias once she is calm:

“And this time I can tell them [the offender] to stop and I’m not going to get mad anymore”.

Children’s reports indicate that their parents are being protective, ensuring they are safe and out of “trouble” by providing guidance on how to keep them from escalating a problem that might lead to more critical harm. Participants’ reports of multi-step processes for managing bullying also indicate parental beliefs that a comprehensive and strategic approach that is culturally relevant and centers positive identity character development is needed to effectively equip and prepare their children to face racism and discrimination.

## 4. Discussion

This innovative study with elementary-school-age children used qualitative data generated from focus groups to identify and center the messages children receive about race and racism. While a larger body of research has focused on experiences of discrimination and identity development among adolescents and adult populations, knowledge of how these processes unfold among younger age groups remains limited. Our findings indicate microaggressions and anti-Black racism from peers to be salient experiences among Black elementary-age children. Our study’s findings reveal Black cultural pride and heritage, and preparation for bias and discrimination to be functionally important lessons Black children learn, providing insight into how RES messages are internalized and interpreted by Black children to cope with race-based bullying. A novel outcome in this study was children’s expressions of emotions as they navigated racial encounters and their assessments of strategies that work and do not work for them. The finding that children attempt to buffer their parents’ racial coping responses (i.e., deciding when and what to share with parents) provides greater evidence that racial socialization is not merely messaging from parents to children but more complex transactions between parents and agentic children navigating race in dynamic contexts. Parents help children develop a positive sense of their own racial–ethnic identity, and children center upon their own perceptions in resisting racist denigration, a concept we call identity framing. The findings here contribute to expanding RES concepts and theory and are important for informing culturally relevant approaches for family-based prevention efforts by including input from the youngest beneficiaries of these interventions, i.e., the children.

Reflecting tenets of Vygotsky’s (1978, 1934/1987) sociocultural theory of cognitive development, caregivers in our study served as more knowledgeable others to their children, teaching them strategies and lessons that were influential in how they navigate racialized encounters. While Vygotsky held that parents delivered their culture and wisdom to children, he did not address how children’s experiences of the world and, in this case, race and discrimination, transform how parents might alter or shift their messages to children. In this study, we find that to help their children cope with adverse experiences of racism, Black parents relay messages to instill a sense of individual uniqueness, perseverance, and cultural pride in their children. Further, children are transactional in the racial–ethnic socialization process by virtue of their ability to intentionally withhold information considered to ignite their parents’ concern or trigger their “over response” as related to race.

When asked about the positives about being Black, identity bolstering, and pride-in-culture comments like being “Black and beautiful,”, and families telling children they are “smart” were reported by participants. Children also indicated that these messages existed in tandem with notes of Black history intended to demonstrate to children that they can successfully navigate discrimination and racial encounters as others have done in the past (i.e., as Ruby Bridges did) to reach their goals. Cultural heritage and pride are core messages in the literature exploring children’s identity and coping (Hughes et al., 2006; D. J. Johnson et al., 2012; E. P. Smith et al., 2003; Stevenson, 1994). Research on RES suggests that Black history and pride messages occur in the recounting of youth, but few studies have used focus groups to extract children’s coping responses. As such, in the current work, we see that children have effectively integrated the information and messages passed onto them by their families to promote personal and Black identity development and strength.

When children were asked about the challenges or difficulties associated with being Black children, they leaned into parental messages centered in preparation for bias when experiencing egregious racial encounters. The children report explicit warnings from their parents about varying types of racial exclusion, experiences where skin color is impactful, and living through invisibility. Children reported their parents’ messages as being quite candid and that the encounters were certain to happen in their lives. Such explicit messages might be linked to sadness in children when paired with their parents’ own degrading experiences of discrimination (Osborne et al., 2021). Yet, there is some indication that these messages are protective if provided in moderation (Harris-Britt et al., 2007; Hughes & Johnson, 2001; Hughes et al., 2006; Yu et al., 2021). Harris-Britt et al. (2007) highlight nuances of how much and when these messages are given to avoid sadness in childhood, suggesting that messages on preparation for bias are best shared later in a child’s development. Rollins (2019) adds further insight with her research on rearing children in biracial families. Parents were desirous of waiting or ignoring race but learned quickly that no preparation seemed more harmful; in essence, they had to get ahead of these unavoidable encounters. Black children in the current study were embedded in multiracial communities and many in multiracial schools, and as such, they developed sophisticated coping responses to overt racial bullying. However, they also expressed a range of emotions at these encounters including anger and sadness.

Preparation for bias was understood by the children as centering themselves in their own integrity or character, holding positive self-confidence/self-esteem, and being responsible for their own behavior and success as protection against the damaging effects of repeated and varied racialized experiences. Coupled with socio-emotional socialization, parents equipped participants with tools for discerning when to ignore and when to confront offenders, with calm but clear communication. Some parents also emphasized the need for children to “handle” matters that became progressively worse. In educational contexts in which Black children are disproportionately disciplined even for minor infractions (Gregory & Fergus, 2017), children in our study described messages from their caregivers less focused upon others, but instead intent on building inner strength and confidence, critical to resistance and thriving.

Specific experiences of discrimination and race-based bullying among participants included derogatory racial name calling (i.e., monkeys and slaves), slights and slurs, and invasion of personal space—unwanted questions, ridicule, and touching of Black hair styles. The egregious referencing to slaves included bullying episodes and demeaning demands to “serve”. In some instances, parental inattentiveness to racialized bullying allowed some situations to eventually become untenable and the children had to be transferred to other schools. Parents provided—and students developed—a variety of coping strategies to navigate what were often frequent assaults of this kind, especially among youth in multiracial school and community settings. The racial coping strategies reported by children were interpersonal, intrapsychic, and relational. Strategies like ignore, retaliate, report incidents to the teacher, and constructive engagement have been found with similar definitions in D. J. Johnson (1988, 2005) and D. J. Johnson et al. (2012). Stevenson et al.’s (2002) *Teenager Experience of Racial Socialization Scale (TERS)* identifies religion or spirituality as a racial coping strategy, a category that emerges in the focus groups described here as well. Children in this study reported drawing upon religion and spirituality as a source of what Yosso (2005) describes as cultural wealth, particularly the areas of navigating and resisting racist encounters. Identity framing is a more unique contribution to the racial coping literature and a complex strategy utilized by children that centers their own perspective of who they are over other’s perceptions. This concept provided by children’s own voices through a focus group approach is congruent with Vygotsky’s schema of private speech (Vygotsky, 1978, 1934/1987) and Sellers et al.’s (1997) notions of prioritizing private over public regard.

In our analysis here, we suggest that a good deal of efficacy exists among children in raising their voices in school settings. Yet, findings from other studies do demonstrate that children’s coping expressions can be suppressed in schools, especially in schools where they are fewer in number, and where school authorities actively perpetuate stereotypic failure narratives, invisibility, and daily racism (Andrews, 2012; Hinds et al., 2022; Stevenson & Arrington, 2012). However, teacher training (Andrews, 2012) and anticipatory support from families (D. J. Johnson et al., 2012) can provide the foundation for greater efficacy in coping with racialized experiences.

In the current study, children not only expressed a range of coping strategies around race-based bullying but also emotions. Stevenson’s (1998) research, touted book *Playing with Anger* (2003), and his book on racial literacy (Stevenson & Stevenson, 2014), discuss children’s expression and witnessing of emotion, anger in particular, when experiencing discrimination. Stevenson (2003) describes a stifled anger in the face of racial encounters among children, with parents having a spill-over or explosive anger in anticipation of micro- and macroaggressions in some contexts. He also reports children’s awareness of their parents and subsequent attempts to buffer or help their parents’ reactions; these included strategies like distraction or withholding information to prevent explosive moments. These transactions all indicate that parents and children alike are being conditioned or required to stifle or reframe their emotions to reduce the risks associated with the interpretation of that anger as a threat in public settings and school. Children in our study expressed “righteous” anger as a core response to discrimination but also a range of other emotions, including sadness, annoyance, and indifference (Florer-Bixler, 2021).

Current work on emotion-focused racial coping is beginning to examine the intersection of socio-emotional development and RES. Dunbar et al. (2017) suggest that adverse effects of preparation for bias might be linked to the suppression of naturally occurring negative emotions such as anger and sadness, in the wake of racial bullying and discrimination. When African American parents allow children to safely express anger and sadness in the safety of their homes, validate their emotions, and equip them to understand when and how to respond, preparation for bias is not linked to the internalizing and externalizing behaviors, found at times in response to this strategy (Dunbar et al., 2022). Moreover, focus group research with parents finds that the quality of the conversation about racial discrimination (e.g., setting, tone, reciprocity) in the contexts of warm family relationships might moderate potential adverse effects upon children’s anxiety and emotions (Coard et al., 2024).

The children in our focus groups described several ways that families validated their emotions and subsequently provided warmth and support that helped them to cope. Children’s *identity framing*—using what they know that is positive about themselves to defend against racialized encounters and microaggressions—is related to other work by Spencer (1983) wherein parents anticipate, and children are destined, to experience dissonance in how they are received and perceived by others, particularly in a White ethos over an ethos that includes and represents them. Parents prepare, and children learn, to elevate their parents’ view of them over and above these external perceptions. Moreover, parents and grandparents built upon messages of cultural pride to inform children’s private speech (Vygotsky, 1978, 1934/1987) in ways that center and frame their racial identity. Congruent with pivotal racial identity research that poses models of private and public regard, parents are building up the private self-perceptions of their children to strengthen and help their children resist and cope with derogatory messages and experiences. Extensive reviews of the literature have found an affirming identity to be protective with outcomes showing increased self-efficacy, self-esteem, emotional adaptation, and academic achievement (Augustine et al., 2022; Rivas-Drake et al., 2014; E. P. Smith et al., 2003; C. Smith et al., 2009; Yu et al., 2021). Children in this study described racial identity support and RES messages about culture and history as aids to bolster themselves when negative racial encounters and bullying occur.

### 4.1. Limitations and Directions for Future Research

The current study represents diverse cultural environments for studying racial socialization and identity development among Black American children in community contexts and extends previous research on RES and racial coping by centering the complex and advanced strategies young Black children learn from caregivers to help focus their thoughts and emotions in the face of oppression. Despite these strengths, as with all research, there are limitations that should be noted. Firstly, although all participants were in middle or late childhood, a core of children represented a particular school context where more identity-centered training and education was promoted each day. In the other focus group settings, children came together from a variety of school settings or were attending predominately White schools. Previous studies indicate that schools are an influential source of information on the meaning of race–ethnicity and culture for youth (Byrd & Legette, 2022; Saleem & Byrd, 2021; Sladek et al., 2022). As such, it is important to acknowledge children’s school contexts in the shaping of their identities and RES experiences.

Secondly, children in our study came from a variety of neighborhoods ranging in racial composition and socio-economic factors and there were several families in our focus groups that were low-income and embedded in an upper-middle class community and school-district. These factors hold some threat for the influence of context on their responses, but also provided a rich tapestry of situations in which families must help their children develop appropriate responses to race and the intersection of race and class where discrimination rears its head (Denise, 2012).

Rather than placing them in a more passive role, focus group research with children encourages their active participation while affirming their voices. As evinced in the current study, centering Black children’s voices in RES and racial coping studies provides a more holistic understanding of these processes that are affecting them directly. Given younger children may not have the skills or capacity to sustain lengthy conversations, particularly around complex topics (Rogers, 1987), it is important that focus groups with children are age-appropriate and use methods that help them communicate their thoughts in ways that feel comfortable. Rules for group discussion were co-created between the participants and facilitators, helping reduce disparities in power between the adult researchers and child participants (Kevern & Webb, 2001). During focus groups, children were given breaks to move around in their seats and, following focus groups, led through stretches and arts-based activities. Previous interview-based studies with Black youth have made use of sensory fidget toys (e.g., Duane, 2023). This may also be a helpful strategy for engaging children in focus group research.

All of our child focus group sessions were conducted in school classrooms, multi-purpose spaces, or youth recreational rooms—environments that were familiar to participants and designed to promote engagement. Yet, conducting focus groups in school settings presents both strengths and limitations. Schools are traditionally understood as spaces where children are encouraged to express their thoughts, making classrooms a familiar and structured setting for data collection. However, for Black children, classrooms can also be sites of silencing, surveillance, and exclusion, where their voices are not always valued or heard in ways that affirm their identities and experiences (Boutte & Bryan, 2021; Gray et al., 2020; Neal-Jackson, 2018). Given this, it is possible that some participants felt constrained in their expression, particularly when discussing racialized experiences within an institutional space that may have contributed to or overlooked such challenges. As such, it is important that future research critically considers how research settings shape participant voice and ensures that methodological choices are attuned to the sociocultural realities of Black children’s school experiences.

Lastly, and importantly, our research focuses upon the experiences of children in middle childhood, an understudied developmental group in the research on RES. Their experiences are profound and valuable; thus, future research should continue to provide a platform to gather their perspectives in ways that are tailored to their developmental needs. Furthermore, research on the transactional processes between parents and children should use approaches that recognize and explore these complex family interactions, as reflected in some of the emerging work with adolescents (Smith-Bynum et al., 2016).

### 4.2. Implications

Children in our focus groups described several ways in which others could help them cope with racialized experiences, including having peers, teachers, and parents who listen to them, understand, and advocate for them. Furthermore, receiving support and coaching in advance on managing these situations was mentioned by these elementary school children as an important way to proactively build their coping strategies. Schools have a transformative role and can enact programs that prepare children in advance to deal with racialized bullying and disciplinary practices that appropriately clarify and execute behavioral consequences. Yet, social history weaves in and out of shifts in school policy that regularly change content. What can schools accomplish when there are constraints of educational policy at the national, state, or local level? For children in independent school settings, families can still help schools shape the agenda and curriculum (Stevenson et al., 2011). Parents advocating for their children with teachers and administrators directly, as a matter of educational outcomes, remains an option even as schools attempt to curtail discussions of race, gender, and other significant parts of youth’s identities. Moreover, families must turn to community-based options like afterschool and church-based programs to achieve positive identity development when schools become less amenable to proven programs for social, political, or economic reasons (Augustine et al., 2022; Ginwright & Cammarota, 2002; Loyd & Williams, 2017; E. P. Smith et al., 2023).

Our study findings also have implications for further developing, adapting, and enhancing family-based preventive interventions for supporting minoritized families. In line with prior scholarship demonstrating the importance of discussing contextual challenges and experiences of discrimination in such interventions (e.g., Coard et al., 2024; Murry et al., 2023; Parra-Cardona et al., 2017; E. P. Smith et al., 2022; Stein et al., 2021), our findings focus on ways families are undertaking such efforts in their parent–child relationships. These insights can be used to further equip and empower caregivers to initiate these important conversations. Importantly, our findings also highlight the extent to which children, even younger children, are hearing these messages and integrating them into their understanding of their experiences and interactions with others and positive racial/cultural identity development (Hughes et al., 2006; Umaña-Taylor & Hill, 2020). However, recent work by Osborne et al. (2021), while demonstrating the positive impact of parental messages emphasizing cultural pride and heritage, shows that experiences of racism and discrimination can overwhelm parental strategies. This again points to the need to dismantle, not restructure, racism and other “isms” at the systemic level. Evidence indicates that addressing the detractors of discrimination in all its forms is among the necessary steps for assuring overall success of all our children. Scholars, in concert with practitioners and policy makers, must continue to proactively address these issues to further promote culturally relevant, equity-based prevention and intervention efforts.

## Data Availability

Anonymized data are available upon request from the corresponding author.
